# Developmental temperature has persistent, sexually dimorphic effects on zebrafish cardiac anatomy

**DOI:** 10.1038/s41598-018-25991-8

**Published:** 2018-05-25

**Authors:** Anastasia Dimitriadi, Dimitris Beis, Christos Arvanitidis, Dominique Adriaens, George Koumoundouros

**Affiliations:** 1 Biology Department, University of Crete, Herakleion, Crete Greece; 2 Developmental Biology, Biomedical Research Foundation Academy of Athens, Athens, Greece; 3 Institute for Marine Biology, Biotechnology and Aquaculture, Hellenic Centre for Marine Research, Heraklion, Crete Greece; 4 Research Group Evolutionary Morphology of Vertebrates, Ghent University, Gent, Belgium

## Abstract

Over the next century, climate change of anthropogenic origin is a major threat to global biodiversity. We show here that developmental temperature can have significant effects on zebrafish cardiac anatomy and swimming performance. Zebrafish embryos were subjected to three developmental temperature treatments (T_D_ = 24, 28 or 32 °C) up to metamorphosis and then all maintained under common conditions (28 °C) to adulthood. We found that developmental temperature affected cardiac anatomy of juveniles and adults even eight months after the different thermal treatments had been applied. The elevation of T_D_ induced a significant increase of the ventricle roundness in juvenile (10% increase) and male (22% increase), but not in female zebrafish. The aerobic exercise performance of adult zebrafish was significantly decreased as T_D_ elevated from 24 to 32 °C. Gene expression analysis that was performed at the end of the temperature treatments revealed significant up-regulation of *nppa*, *myh7* and *mybpc3* genes at the colder temperature. Our work provides the first evidence for a direct link between developmental temperature and cardiac form at later life-stages. Our results also add to the emerging rationale for understanding the potential effects of global warming on how fish will perform in their natural environment.

## Introduction

Climate change poses a great threat to global biodiversity, affecting the physiology of species^1^ and the distribution of populations^2^. Thus, it is of crucial importance to better understand the physiological and molecular mechanisms underlying the responses of organisms to fluctuating environments^3–5^. Interest in phenotypic plasticity, i.e. the ability of individual genotypes to produce different phenotypes when exposed to different environmental conditions, has grown exponentially during the last decades in an effort to understand the physiological responses of organisms to global warming^6^. Still, despite the intensifying interest on temperature as a major abiotic factor determining species distribution^7,8^, there is relatively little knowledge concerning the mechanisms underlying thermal plasticity during development and how they influence the success and fitness of different species^9^.

Fish constitute a highly plastic group of ectotherms, exhibiting a great ability to alter their phenotype in relation to challenging environments^10,11^. Previous research has identified that within the zone of tolerance, temperature affects fish performance at a variety of levels of biological organization^11,12^. Developmental temperature has already been recognized as an acute regulating factor in sex determination^13,14^, body shape^15,16^, developmental pattern^17,18^, muscle enzyme activity^19^, as well as muscle cellularity^20,21^, swimming performance^22,23^, thermal acclimation capacity^24^,stress and immune responsiveness^25^ of fish. Despite the link of cardiac anatomy to maximum cardiac output and critical swimming speed^26–28^, published data have primarily focused on the effects of temperature on heart function and ontogeny^29–31^, as well as on the mechanisms underlying the preservation of cardiac function across seasonal temperature fluctuations^32–35^. To our knowledge, the plastic responses of the fish cardiovascular system to developmental temperature remain unexplored.

We hypothesized that exposure to altered temperatures during early development could persistently influence fish cardiac anatomy and ultimately, the cardiac output of juvenile and adult fish. To test our hypothesis, we used the zebrafish as a model to study thermally induced phenotypic plasticity. In the wild, zebrafish experience wide seasonal temperature fluctuations from as low as 6 °C in winter to over 38 °C in summer^36^. We reared zebrafish embryos and larvae in three different developmental temperatures (24, 28 and 32 °C, lying within the natural thermal range for this species)^36^ up to metamorphosis and then we exposed them at a common temperature (28 °C) till adulthood. We found that developmental temperature has striking but variable effects on zebrafish cardiac anatomy and swimming performance, even several months after the end of fish exposure to the different thermal regimes.

## Results

### Developmental temperature affects cardiac morphology in males and early juveniles

**Figure 1:**
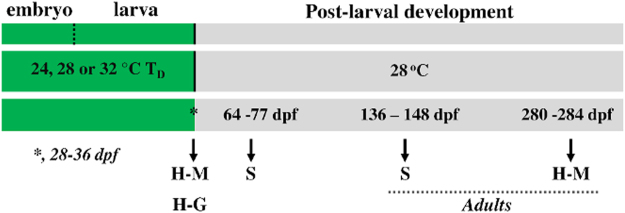
Experimental design of the study. Fish were subjected to one of three developmental temperature (T_D_) treatments up to metamorphosis (*) and then at a common temperature till adulthood. Transcriptomic analysis of the heart (H-G) was performed 1 d before the transfer of the fish to the same temperature. Morphometric analysis of the heart (H-M) was performed 1 d before the transfer of the fish to the same temperature (early juveniles) and at the adult stage (9–10 months later). Swimming performance (S) was measured 4 weeks after the transfer of the fish to the same temperature (late juveniles) and at the adult stage (3–4 months later). T_D_, developmental temperature; dpf, days after fertilization.

**Figure 2:**
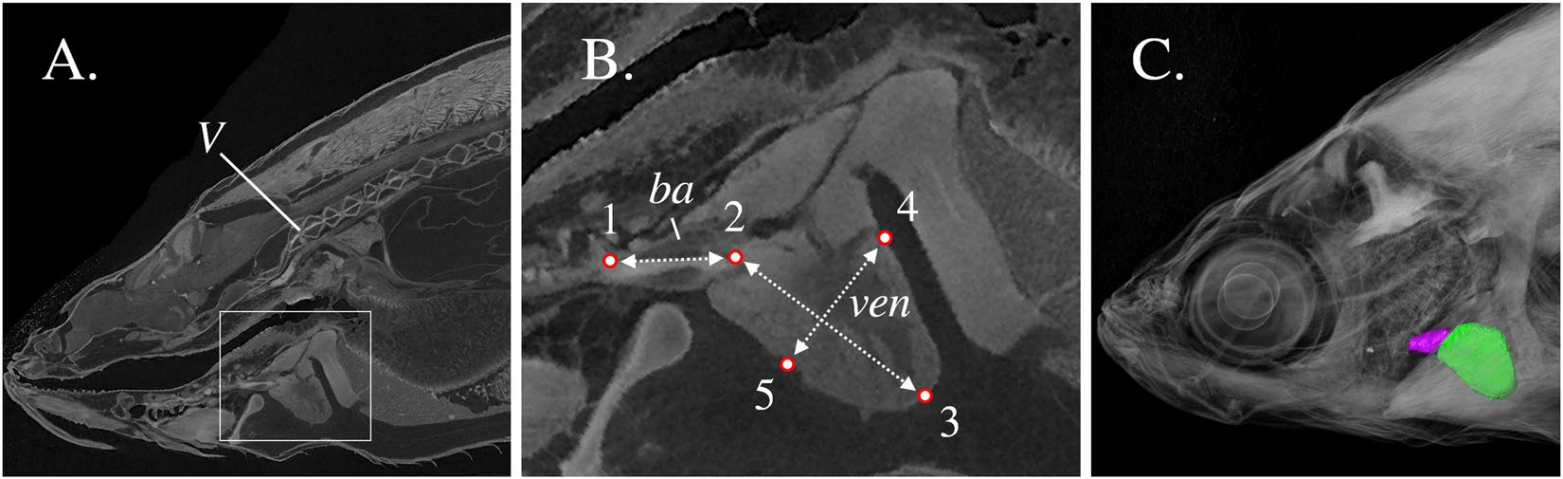
Multiple views from a single scan of an adult zebrafish. (**A**) Representative oblique slice defined by three landmarks (anterior and posterior end of the bulbus arteriosus, the centre of the 1st vertebra). (**B**) Inset of Fig. 2A, showing the ventricle (ven), bulbus arteriosus (ba), as well as the distance measurements taken. (**C**) Three-dimensional volume rendering of the bulbus arteriosus (purple) and ventricle (green). V, first vertebra. ba, bulbus arteriosus. ven, ventricle. Landmark 1, bulbus junction with the first branchial arch. Landmark 2, ventricle – bulbus valve. Landmark 3, ventricle apex. Landmarks 4 and 5 define the widest distance of the ventricle, perpendicularly to ventricle length (D_2–3_). D_1–2_, Bulbus-arteriosus length (BaL); D_2–3_, Ventricle length (VL); D_4–5_, maximum ventricle depth (VD), perpendicular to VL.

Morphology of the juvenile and adult hearts was assessed at the end of the exposure to the different temperatures (early juvenile stage, definitive pigmentation patterns on the caudal and anal fins) and 8 months later (adult stage), by micro-ct imaging (Materials and Methods, Figs 1 and 2). Ventricular shape was measured as the length-to-depth ratio (VL/VD). Ventricular volume and bulbus arteriosus length were standardized to SL (VeV/SL and BaL/SL).

**Figure 3:**
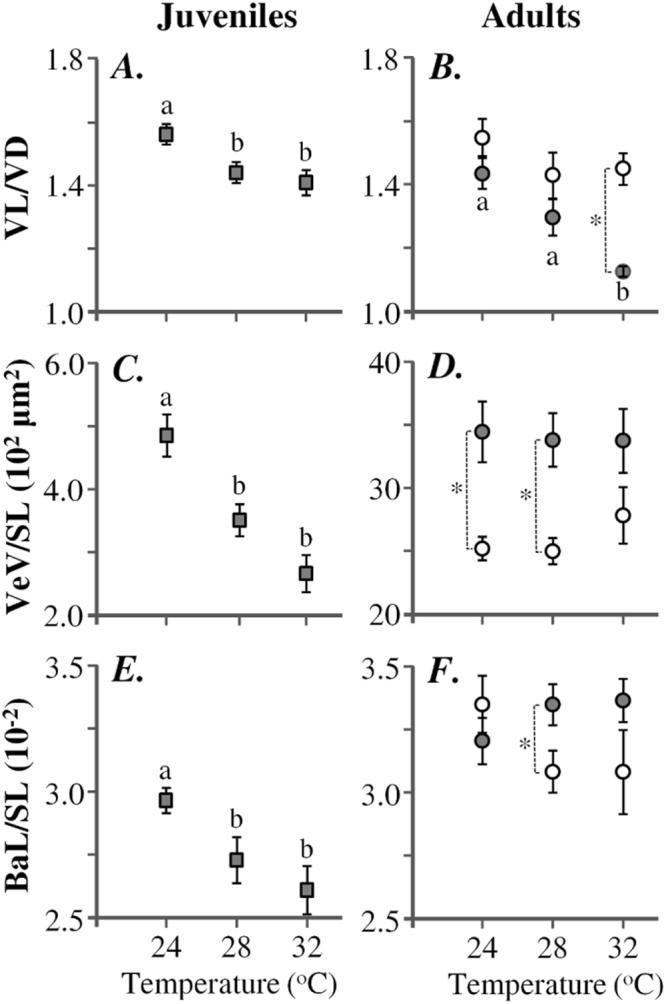
Changes in early juvenile and adult cardiac anatomy in response to developmental temperature. Cardiac morphometric indices in males (filled circles, n = 7–9), females (open circles, n = 7–9) and early juveniles (squares, n = 8–15) of different treatment groups. (**A**,**B**) Ventricle length-to-depth ratio, (**C**,**D**) ventricle volume normalized to standard length, (**E**,**F**) bulbus arteriosus length normalized to standard length. BaL, bulbus arteriosus length. SL, standard length. VD, ventricle depth. VL, ventricle length. VeV, ventricle volume. Values without a letter in common are statistically different (p < 0.05, Kruskal-Wallis and Mann-Whitney U test). Asterisks indicate significant statistical differences (p < 0.05, Mann-Whitney U test) between males and females of the same treatment. Error bars equal to ± 1 SEM.

**Figure 4:**
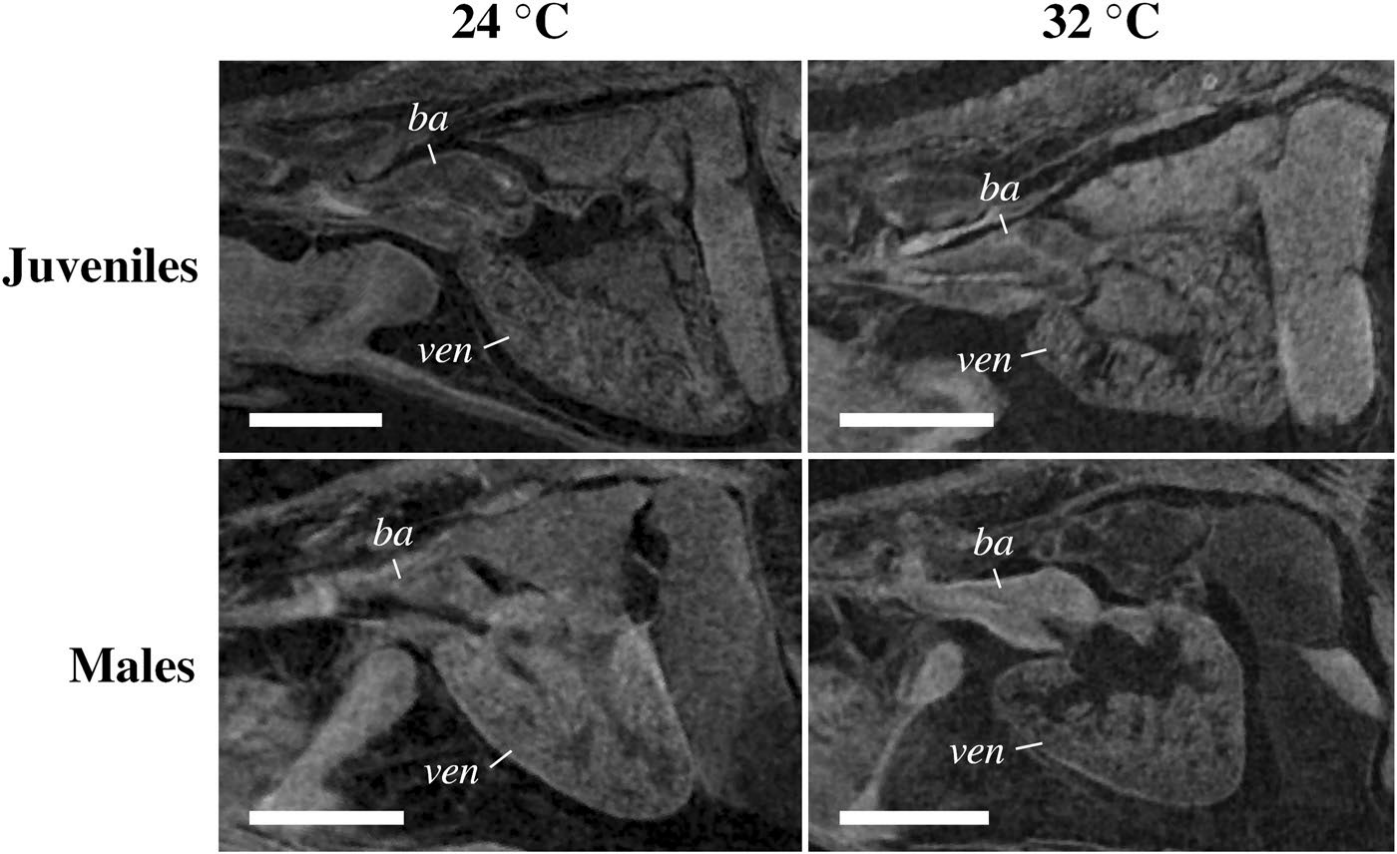
Representative primary images showing the comparatively more round ventricle in juvenile and male zebrafish reared at 32 °C developmental temperature (T_D_). Scale bars equal to 0.25 (juveniles) or 1.0 mm (males). ba, bulbus arteriosus. ven, ventricle.

Water temperature during the embryonic and larval period (further referred to as T_D_) significantly affected the ventricular shape of juvenile and male, but not of female zebrafish (Fig. 3A,B). In males, the shape index VL/VD significantly decreased with the elevation of T_D_, from 1.43 ± 0.05 at 24 °C to 1.12 ± 0.02 at 32 °C (p < 0.05, Mann-Whitney U test), revealing a comparatively rounder ventricle at higher T_D_ (Figs 3B and 4). A similar effect of T_D_ on ventricular shape was observed in juvenile zebrafish, with the group of 24 °C presenting a significantly bigger VL/VD ratio (1.56 ± 0.03) than the group of 32 °C (1.40 ± 0.04, p < 0.05, Mann-Whitney U test) (Fig. 3A). In adult fish, T_D_ had no significant effect on the size of the ventricle (VeV/SL, p > 0.05, Kruskal-Wallis test, Fig. 3D) and of the bulbus arteriosus (BaL/SL, p > 0.05, Kruskal-Wallis test, Fig. 3F). In the early juveniles, both VeV/SL and BaL/SL significantly decreased with the elevation of T_D_ (p < 0.05, Kruskal-Wallis test) (Fig. 3C,E).

Sexual differences were significant on a T_D_ dependent level. Compared to the females, male zebrafish presented a significantly smaller ventricular shape index (VL/VD) at 32 °C T_D_ (p < 0.05, Mann-Whitney U test) but not at the other thermal conditions tested (Fig. 3B). Ventricle size was significantly bigger in males than in females at all but 32 °C T_D_ tested (Fig. 3D). Male zebrafish presented a significantly longer bulbus arteriosus than female, but only at 28 °C (p < 0.05, Mann-Whitney U test) (Fig. 3F). Compared to the early juveniles, adult zebrafish presented significantly larger ventricle and bulbus arteriosus standardized sizes, at all the experimental conditions (p < 0.05, Mann-Whitney U test, Table S1). No differences were observed in the ventricular shape between the females and early juveniles (p > 0.05, Mann-Whitney U test, Table S1). At 28 and 32 °C T_D_, the shape index VL/VD was significantly smaller in male than in the early juveniles (p < 0.05, Mann-Whitney U test, Table S1).

### Developmental temperature modifies the expression levels of cardiac remodeling markers

**Figure 5:**
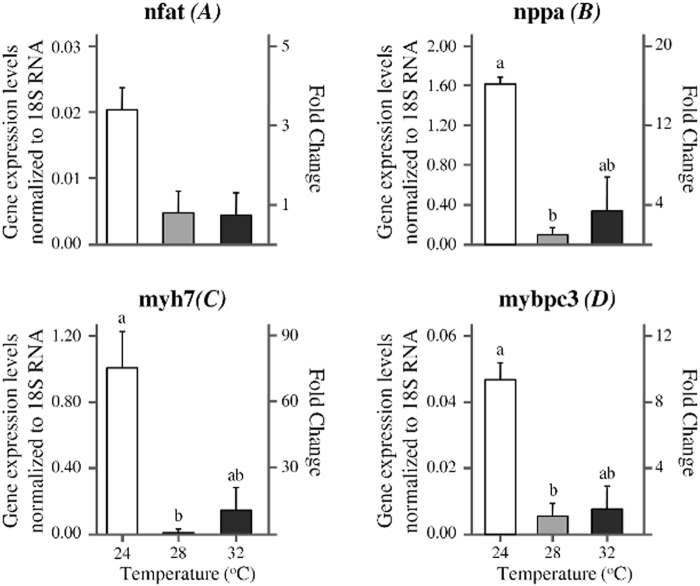
Effect of developmental temperature on the relative gene expression levels (mRNA). (**A**) Nuclear factor of activated T cells (*nfatc1*), (**B**) natriuretic peptide precursor a (*nppa*), (**C**) myosin heavy chain 7 (*myh7*) and (**D**) myosin binding protein C (*mybpc3*) in juvenile zebrafish at the end of the thermal treatment application. Expression levels were measured by real-time PCR using SYBR green and normalized to 18S RNA internal reference gene. Fold change is relative to 28 °C controls. n equals to 4, 4 and 3 for 24, 28 and 32 °C group respectively. Values without a letter in common are statistically different (p < 0.05, Kruskal-Wallis and Mann-Whitney U test). Error bars equal to 1 SEM.

In order to track down the early molecular modifications taking place at the end of the different thermal treatments, we examined the expression levels of four markers for cardiac remodeling, *nfatc1*, *nppa*, *myh7* and *mybpc3*. Developmental temperature had a significant effect on the relative expression levels of *nppa*, *myh7* and *mybpc3* (p < 0.05, Kruskal-Wallis test), but not of the *nfatc1* (p > 0.05, Kruskal-Wallis test) (Fig. 5). No significant differences were detected between early juveniles of 28 and 32 °C T_D_. Compared to the fish of 28 °C T_D_, fish of 24 °C T_D_ presented a 16, 74 and 9-fold increase of *nppa*, *myh7* and *mybpc3* levels respectively (Fig. 5B–D).

### Changes in cardiac morphology of male zebrafish are associated with altered swimming performance at the different temperature regimes

**Figure 6:**
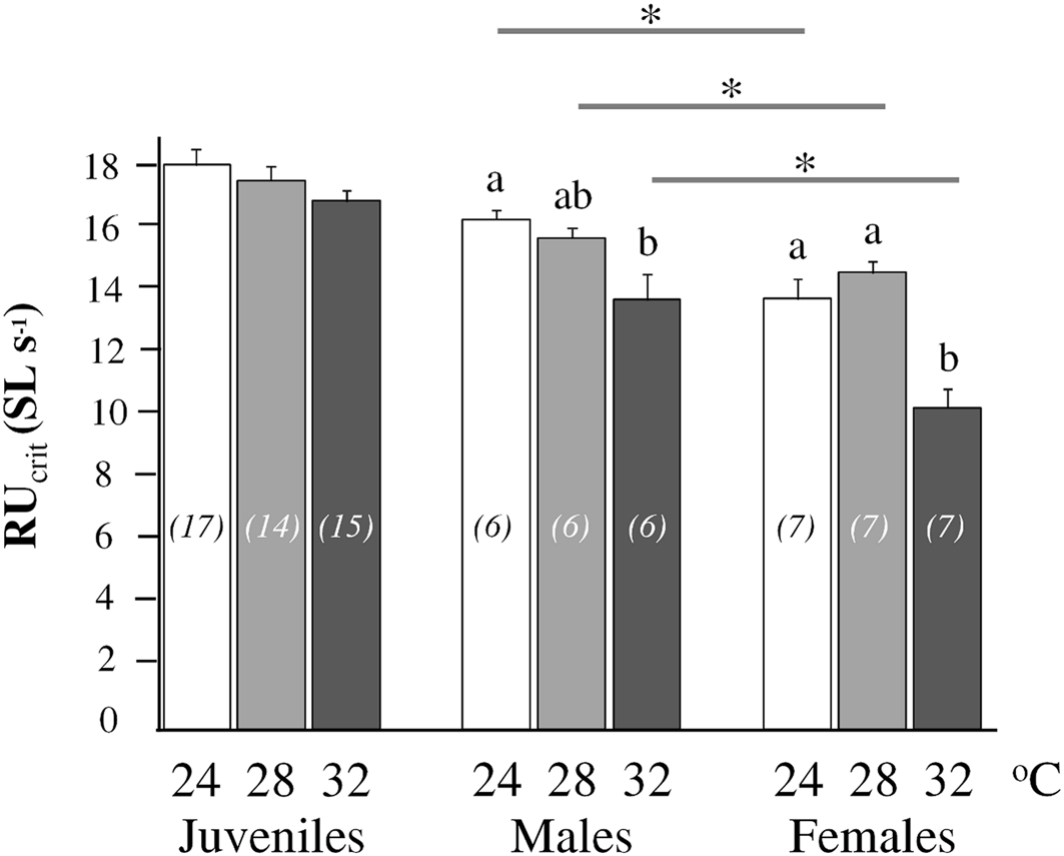
Effect of developmental temperature on the aerobic swimming performance of zebrafish. Mean RU_crit_ was measured in late juveniles (n = 15–17), males (n = 6) and females (n = 7) of different treatment groups. Values without a letter in common are statistically different (p < 0.05, Kruskal-Wallis and Mann-Whitney U test). Asterisks indicate significant statistical differences between males and females of the same developmental temperature (p < 0.05, Mann-Whitney U test). Error bars equal to 1 SEM.

The relative critical swimming velocities measured for males, females and late juveniles of the three different thermal treatments are depicted in Fig. 6. Developmental temperature and sex significantly affected zebrafish swimming performance. Although temperature had no significant effect on RU_crit_ values of zebrafish juveniles (p > 0.05, Kruskal-Wallis test, Fig. 6), males reared at 24 °C T_D_ achieved significantly higher swimming velocities than those raised at 32 °C T_D_ (p < 0.05, Mann-Whitney U test, Fig. 6). Similarly, females raised at 32 °C T_D_ had decreased RU_crit_ values compared to the 28 and 24 °C groups (p < 0.05, Mann-Whitney U test, Fig. 6). Sex had a significant effect on zebrafish swimming performance, with males achieving higher swimming velocities than females at all thermal treatments (p < 0.05, Mann-Whitney U test, Fig. 6).

## Discussion

Consistent with our initial hypothesis we found that the temperature during development has persistent effects on zebrafish cardiac anatomy and on swimming performance. Despite the significance of water temperature for the cardiac function in developing fish^29,30^, to our knowledge, this is the first study demonstrating the programming of cardiac shape by the temperature which was experienced by the fish during embryonic and larval stages. We showed that differences in developmental temperature could induce persistent changes of cardiac shape and decrease the aerobic performance of the males (i.e. 22% decrease of the VL/VD ratio, 16% decrease of the RU_crit_). In support to our results, previous studies also demonstrate that ventricular roundness is linked to an inferior cardiac pumping capacity and critical swimming speed. In rainbow trout, poor swimming (27% decrease in U_crit_) was linked to a more round ventricle (13% decrease in VL/VD) and a lower maximum cardiac output (by 30%)^26^. In zebrafish, the sublethal exposure of embryos to crude oil induced an increase of the ventricular roundness (9% decrease in VL/VD) and a decreased aerobic performance (18% decrease in RU_crit_) in adults^28^.

The acquisition of the shape of organs during ontogeny is a complex process, during which shape may be sculpted by cell proliferation, cell movement, as well as by changes in cell shape and size, under the influence of the local environment. Cardiac development is a paradigm where the interplay between the form and function during organogenesis could be studied. During ventricle emergence in zebrafish for example, ventricular shape is controlled by regionally confined cardiomyocyte shape and size changes, which are regulated by extrinsic physical forces (e.g. blood flow) and intrinsic cellular properties (e.g. contractility)^37^. In the present study, the programming of ventricular shape (VL/VD) by developmental temperature might be attributed to the temperature effects on the heart contraction rate and hemodynamic forces^30,38,39^, which in turn modulate major events in heart morphogenesis (e.g. chamber emergence, valvulogenesis, ventricular trabeculation)^40–42^. In the case of zebrafish, over the temperature range of 25–31 °C, Q_10_ for heart rate is constant and equal to 1.2–2.5 throughout development^30^.

Following our results, at the end of fish exposure to the different thermal regimes (early juvenile stage), the relative ventricular volume (VeV/SL) and bulbus arteriosus length (Bal/SL) decreased as temperature increased from 24 to 32 °C. During the next period of acclimation to a common temperature (28 °C), however, the initial between group differences in VeV/SL and Bal/SL disappeared (Fig. 3D,F). It is therefore concluded that, in contrary to the persistent non-reversible changes of ventricular shape in male fish (VL/VD), the changes of Bal/SL and VeV/SL are reversible acclimation responses of the heart to temperature. In support to our findings, previous studies demonstrate that thermal acclimation causes a substantial cardiac remodelling, across multiple levels of biological organisation, as a mechanism of fish to maintain efficient cardiac function across seasonal temperature changes^43^. Concerning the ventricle size, a number of studies demonstrate that cold acclimation induces a significant increase in relative ventricular mass, through a mechanism involving myocyte hypertrophy and hyperplasia^43^. To our knowledge, no studies exist on the acclimation responses of the bulbus arteriosus. As the bulbus arteriosus serves the dampening of the ventricular pulse pressure and the maintenance of a positive pressure for arterial run-off during the diastole of the ventricle^44^, the increase of its relative length at 24 °C T_D_ (BaL/SL, Fig. 3E) might be related to the observed increase of the relative ventricular volume (VeV/SL, Fig. 3C).

In order to decipher the early molecular changes taking place at the end of the application of the different temperatures (early juveniles), we examined the expression levels of four established marker genes for myocardial remodelling. Specifically, we studied the expression levels of *nppa* (natriuretic peptide precursor a), a marker of cardiomyocyte proliferation^45^ and chamber differentiation during cardiogenesis^46^, *nfat* (nuclear factor of activated T cells), a crucial transcription factor involved in hypertrophic mechanisms in mammalian systems^47^, as well as of *myh7* (myosin heavy chain 7) and *mybpc3* (myosin binding protein C) structural genes regulating cardiac contractility and relaxation^48,49^. Our results demonstrated that the expression of the three out of four examined genes (*nppa*, *myh7*, *mybpc3*) was significantly up-regulated at the lower temperature tested. As at the juvenile stage all the basic morphological features of zebrafish heart have been finalised^50,51^, this gene up-regulation is expected to be associated with cardiac acclimation responses, rather than with the programming of ventricular shape. Similarly to our results, in female rainbow trout, cold acclimation induced a significant up-regulation of gene markers of muscle growth (e.g. ventricular myosin heavy chain, natriuretic peptide A) and of collagen regulator genes, without however any significant alterations in ventricular mass^34^.

In the present study we showed that male zebrafish have larger relative ventricle size (VeV/SL) than females (Fig. 3D). As many other fish species, zebrafish is characterised by a strong sexual dimorphism in relation to body shape (swollen belly of females vs slender body of males), behaviour and metabolism^16,36,52^, as well as in diastolic ventricular function^53^ which might be linked to differences in swimming modes and other aspects of their life habits (e.g. energy allocation trade-offs with egg production, etc.). In accordance to our results, in salmonids and other fish species, males undergo a cardiac enlargement during reproductive maturation, stimulated by sex steroids and resulting in a comparatively larger heart than in females^33^.

In addition to the sexual dimorphism in VeV/SL, our results show that zebrafish is characterised by different reaction norms of ventricular shape (VL/VD) to developmental temperature (T_D_). In contrary to the significant plastic response of males, female zebrafish exhibited a limited plasticity in ventricular roundness in response to T_D_. Sexual dimorphism in cardiac responses to temperature is also evident in salmonids, where cold acclimation induces significant changes in ventricular size and connective tissue content of male, but not of female fish^35^. In the present study, it might therefore be expected that the plastic response of female aerobic capacity to T_D_ (Fig. 6) is associated with changes in other features which are known to affect fish swimming performance (e.g. body shape in relation to hydrodynamics, properties of swimming muscles, energy metabolism, mitochondria number)^23,24,54^. In future, it would be interesting to examine the relative contribution of these features and of heart anatomy on the thermally induced plasticity of the swimming performance of both male and female zebrafish. In a similar way, such an integrated approach might explain the lack of significant differences in the swimming performance of the different T_D_ groups at the juvenile stage.

Critical swimming speed has been widely used to assess the environmental effects on fish aerobic performance^24,26,28,55,56^. Our data show that rises in water temperature at critical early life stages can have detrimental effects on the physical capacity of adult fish. Given that aerobic swimming capacity has vital ecological significance (i.e. for migration, routine swimming and foraging)^57,58^ our findings have relevance to fish populations and the concerns of global warming and seasonal temperature perturbations^1,7^. Besides fish, developmental temperature induces highly plastic responses also in other ectotherm vertebrates^59–61^. Our results may therefore have implications beyond fish, relating to potential impacts of developmental temperature on the heart shape of amphibians and reptiles.

## Materials and Methods

### Experimental treatments

The overall experimental design is depicted in Fig. 1. Blastula-stage embryos were reared until metamorphosis (Table S2) at 23.9 ± 0.3, 27.8 ± 0.3, or 31.5 ± 0.5 °C water temperature (mean ± SD, temperatures lying within the natural thermal range for this species)^36^. Subsequently, all groups were raised to adulthood at 28.0 ± 0.2 °C.

The same tenth-generation fish population, composed of 125 males and 125 females, was used as broodstock for all experiments. Breeders were kept in one 100 L aquarium, connected to a closed recirculation system, at 28.0 °C (±0.5 °C), 500–700 μS/cm conductivity, 7.0–7.5 pH, 85–95% oxygen saturation and 14/10 h light/dark photoperiod. Adult zebrafish were fed on commercial flakes (Cichlid Omni Flakes, Ocean Nutrition Europe, Essen, Belgium) and bloodworms. Fertilized eggs were collected 2–3 h after spawning and were gradually acclimated to the treatment temperatures of 24 or 32 °C (at a rate of 1 °C/h). Egg incubation and larval rearing were performed in 9 L aquaria (33 eggs/L initial density) connected to closed recirculation systems, at 7.0–7.5 pH, 500–700 μS/cm conductivity, 85–95% oxygen saturation, 14/10 h light/dark photoperiod and a water exchange rate of 100% of the aquarium volume per hour. At 12–14 mm SL, fish were transferred to 8 L aquaria, at a stocking density of 8 individuals L^−1^. Larvae were fed on newly hatched *Artemia* nauplii five times per day. After 12–13 mm SL, commercial diet was provided to the experimental populations (Cichlid Omni Flakes, Ocean Nutrition Europe, Essen, Belgium), and provision of *Artemia* nauplii was gradually terminated.

Heart morphometry and transcriptomic analyses were performed for each developmental temperature (T_D_) group, 1 d before the transfer of the fish to the same temperature (28 °C). Heart morphometry was repeated at the adult stage, 9–10 months later (Fig. 1, Tables S3 and S4). Transcriptomic analysis focused on the expression levels of genes involved in cardiac remodeling, such as *nppa* (natriuretic peptide precursor a), *nfatc1*, (nuclear factor of activated T cells), *myh7* (myosin heavy chain 7) and *mybpc3* (myosin binding protein C)^46–49^. Swimming performance was estimated in each experimental group, four weeks after the transfer of the fish to the same temperature (late juveniles) and at the adult stage (Fig. 1). All trials were performed in duplicate, with embryos randomly obtained from two different spawns of wild-type zebrafish breeders. Two sets of identical experimental protocols were conducted, each with two replicates, where zebrafish embryos were raised and treated likewise till adulthood. Heart morphometry and gene expression were studied in the first set of experiments. Swimming performance was assessed in the second set of experiments to specify the effect of developmental temperature on zebrafish aerobic capacity.

### Micro-CT imaging

Eight to fifteen individuals per experimental condition for the early juveniles (Table S3) and 7–9 individuals per sex and experimental condition for the adults (Table S4) were euthanatized with an overdose of buffered tricaine methanesulfonate (MS222), photographed and measured for standard length (SL, tip of snout to the base of the central caudal-fin ray, TpsDIG2 software). Specimens were fixed in 5% phosphate buffered formalin, stained for six days with 2.5% phosphomolybdic acid (PMA, a contrast agent for soft tissue discrimination)^62^, and dehydrated in 70% ethanol. Stained fish were individually scanned (SkyScan 1172, 3–5 μm resolution, 180° total rotation, 1450 ms exposure time, 50 kV voltage, 199 μΑ). During scanning, specimens were kept in ethanol-saturated vials to prevent shrinkage from dehydration. Projection images obtained during the scanning process were reconstructed (NRecon software, SkyScan) into cross sections and stored as TIFF image stacks, enabling the processing of image data. The TIFF virtual images were imported in the software Amira v.5.2 (Visage Imaging, Berlin, Germany, Burlington USA) so as to obtain a two- and three-dimensional representation of the cross-sectional image data. Two-dimensional morphometrics were taken on a standardized oriented sagittal plane defined by (a) the anterior end of bulbus arteriosus, (b) the posterior end of bulbus arteriosus and (c) the centre of the 1st vertebra (Fig. 2A,B, Oblique Slice Module, Amira). The selected approach of plane definition standardized the imaging and measurement methodology of the heart ventricle and bulbus arteriosus.

For heart morphometry, the xyz coordinates of five distinct landmarks of the bulbus arteriosus and the ventricle were retrieved by means of Amira (Fig. 2B). Ventricle length (VL) was determined as the distance between ventriculo-bulbar valve (landmark 2) and the apex (landmark 3), and bulbus-arteriosus length (BaL) as the distance between the base of the posterior aortic arch (landmark 1) and the ventriculo-bulbar valve (landmark 2) (Fig. 2B). Ventricle depth (VD) was determined as the widest distance of the ventricle, perpendicularly to ventricle length (landmarks 4 and 5) (Fig. 2B). Moreover, as proposed by Hicken and co-authors^28^, the length-to-width ratio was chosen as a suitable index for the ventricular shape. Three-dimensional analysis of the zebrafish heart included estimations of the ventricular volume (VeV), after a semi-automatic segmentation based on grey-scale values of the voxels (Amira segmentation editor). Measurements were made blind to treatment group. The effect of developmental temperature on zebrafish heart morphology was tested by means of the non-parametric Kruskal-Wallis test (α = 0.05). In the case of a significant effect, post hoc mean comparisons were performed by means of Mann-Whitney U test (α = 0.05).

### Swimming performance

Swimming performance was assessed by estimating the sustained critical swimming speed (U_crit_) with a custom-designed swimming apparatus with a swim tunnel of 70 cm length, 10 cm depth and 5 cm width^23^. Different flow regimes were obtained by the use of external magnetic pumps and adjustable valves. Water speed in the swim tunnel was calibrated by means of an electromagnetic flow-meter (Valeport, Model 801). Plastic straws helped in maintaining laminar flow through the swimming channel and in preventing fish from forward-escape.

Swimming trials were performed separately for each specimen. Eighteen to twenty hours prior to the swimming tests, experimental fish were transferred to one holding aquarium and deprived of food. For the swimming trials, fish of similar size were placed in the swimming tunnel for 10 min at 2 SL s^−1^ water velocity. Then, water velocity increased every 10 min at a rate of 1 SL s^−1^. Fatigue was determined when fish left the swimming channel, unable to react to visual or acoustic stimuli from the side or behind^23^. U_crit_ was calculated according to the formula U_crit_ = U_i_ + (U_ii_·t_i_/t_ii_), where U_i_ is the highest swimming velocity (mm s^−1^) maintained for a full interval of 10 min, U_ii_ the velocity increment (1 SL s^−1^), t_i_ is the time interval that each individual swam at the fatigue velocity, and t_ii_ is the time interval between velocity changes (i.e. 10 min)^63^. Water temperature was maintained at 28.0 °C and oxygen saturation at 100%.

Fatigued fish were anaesthetized (MS222, 100 mg/L^−1^), measured for SL, and examined for the presence of gross morphological abnormalities. Only fish with normal morphology were included in the analysis. The swollen belly of female fish was used to distinguish sex among individuals^16^. In total, 14–17 individuals per experimental condition for the late juveniles (7–9 per replicate, Table S5) and 6–7 individuals per sex and experimental condition for the adults (2–5 per replicate, Table S6) were subjected to swimming tests. Relative critical swimming speed (RU_crit_) was calculated as the ratio of U_crit_ to the SL of each specimen. The effect of developmental temperature on juvenile and adult RU_crit_ was tested by means of the non-parametric Kruskal-Wallis test (α = 0.05). In the case of a significant effect, post hoc mean comparisons were performed by means of Mann-Whitney U test (α = 0.05).

### Gene expression analysis

From each thermal regime, a sample of zebrafish early juveniles was anaesthetized, measured for SL and subjected to heart dissection (Fig. 1). Each heart was homogenized in 50 μl TRIzol reagent (Invitrogen), snap frozen in liquid nitrogen and stored in a −80 °C freezer. Two to eight individually dissected hearts per experimental condition (equally taken from each experimental replicate) were pooled to constitute one sample. Three to four samples were tested for each experimental condition.

Primer sequences and gene accession numbers are depicted in Table S7. Primers were designed using Primer3 software (http://bioinfo.ut.ee/primer3/). Total RNA was isolated with TRIzol reagent (Invitrogen Life Technologies, Calsbad, CA, USA), purified by Turbo DNase (Ambion) and reverse transcribed using PrimeScript RT reagent kit (Takara Bio Inc, Shiga, Japan). For the quantification of gene expression levels, real-time quantitative PCR (Q-PCR) was performed. cDNA was diluted 1:8 for all target genes, including the reference gene. Reactions contained 10 μl οf KAPA SYBR Fast qPCR Kit (Kapa Biosystems), 300 nM concentration of forward and reverse primers and 5 μl of cDNA. Reactions were run in a Light Cycler 96 (Roche, Germany) real-time PCR machine using the following 2-step protocol: 2 min at 50 °C, 10 min at 95 °C, followed by 40 cycles of 15 sec at 95 °C and 60 sec at 60 °C, and a melting curve analysis of 60 sec at 95 °C, 60 sec at 65 °C and 10 sec at 95 °C. Reactions without template were used as negative controls. 18 S RNA gene was used as an internal control. Samples run in duplicate and fluorescence was measured to estimate the values of the threshold cycles (Ct).

Ct values were normalized for each gene to the housekeeping gene: 18S RNA. Expression levels were calculated by the average 2^−ΔCt^ values^64^ where the ΔCt was determined by Eq. (1)

1 $${\rm{\Delta }}\text{Ct}=({{\rm{C}}}_{{\rm{t}},{\rm{target}}{\rm{gene}}}\mbox{--}{{\rm{C}}}_{{\rm{t}},{\rm{reference}}{\rm{gene}}})$$

Fold change in the target genes was calculated for each group by normalizing the 2^−ΔCt^ average values to the 28 °C 2^−ΔCt^ average value. All statistical analyses were tested by means of the non-parametric Kruskal-Wallis and Mann-Whitney U tests (α = 0.05).

### Ethics statement

All the experimental procedures involving animals were performed in accordance with Greek (PD 56/2013) and EU (Directive 63/2010) legislation for animal experimentation and welfare. All protocols were approved by the Animal Care Committee of the Biology Department of the University of Crete (Permit Number: 3628/17).

### Data availability

All data generated and analysed during this study are included in this published article (and its Supplementary Information files).

## Electronic supplementary material

Supplementary information

Dataset 1
