# Supplementary material for: Developmental temperature has persistent, sexually dimorphic effects on zebrafish cardiac anatomy
Source: Sci Rep. 2018 May 25;8:8125. doi: 10.1038/s41598-018-25991-8 (PMC5970236; doi:10.1038/s41598-018-25991-8)
Supplement: Supplementary file 1 — Supplementary information [file 41598_2018_25991_MOESM1_ESM.doc]

**Developmental temperature has persistent, sexually dimorphic effects on zebrafish cardiac anatomy**

Anastasia Dimitriadi^1^, Dimitris Beis^2^, Christos Arvanitidis^3^, Dominique Adriaens^4^, George Koumoundouros^1*^

**1** Biology Department, University of Crete, Herakleion, Crete, Greece

**2** Developmental Biology, Biomedical Research Foundation Academy of Athens, Athens, Greece

**3** Institute for Marine Biology, Biotechnology and Aquaculture, Hellenic Centre for Marine Research, Heraklion, Crete, Greece

**4** Research Group Evolutionary Morphology of Vertebrates, Ghent University, Gent, Belgium

*, corresponding author, [gkoumound@uoc.gr](mailto:gkoumound@uoc.gr)

**Table S1.** Effects of ontogenetic stage on cardiac morphometric indices.

| T_D_ (°C) | Juveniles | Males | Females | p(J-m) | p(J-f) |
| --- | --- | --- | --- | --- | --- |
| **VL/VD** | | | | | |
| 24 | 1.557±0.0330 (15) | 1.433±0.0490 (9) | 1.547±0.0593 (9) | - | - |
| 28 | 1.435±0.0326 (10) | 1.296±0.0583 (9) | 1.428±0.0714 (8) | * | - |
| 32 | 1.404±0.0409 (8) | 1.123±0.0175 (8) | 1.448±0.0510 (7) | * | - |
| **VeV/SL (10^2^ μm^2^)** | | | | | |
| 24 | 4.827±0.3275 (15) | 34.467±2.3729 (9) | 25.175±0.9197 (9) | * | * |
| 28 | 3.487±0.2516 (10) | 33.808±2.1431 (9) | 25.001±1.0156 (8) | * | * |
| 32 | 2.652±0.2944 (8) | 33.787±2.5177 (8) | 27.825±2.2039 (7) | * | * |
| **BaL/SL** | | | | | |
| 24 | 0.029±0.0005 (15) | 0.032±0.0009 (9) | 0.033±0.0011 (9) | * | * |
| 28 | 0.027±0.0009 (10) | 0.033±0.0008 (9) | 0.030±0.0008 (8) | * | * |
| 32 | 0.026±0.0009 (8) | 0.033±0.0008 (8) | 0.030±0.0017 (7) | * | * |

T_D_, developmental temperature; VL/VD, ventricle length-to-depth ratio; VeV/SL, ventricle volume, standardized to standard length; BaL/SL, bulbus arteriosus length standardized to SL. Values are means ± SE (n). Asterisks indicate the significance of the post hoc mean comparisons between juveniles and males [p(J-m)] or females [p(J-f)] (p<0.05, Mann-Whitney U test).

**Table S2.** Age and mean standard length of zebrafish juveniles at the end of the application of the three different temperature conditions.

| T_D_ (°C) | Dpf | R1 | |  | R2 | |
| --- | --- | --- | --- | --- | --- | --- |
|  |  | n | SL (mm) |  | n | SL (mm) |
| 24 | 35 | 30 | 13.9 ±1.8 |  | 30 | 12.9±1.4 |
| 28 | 28 | 20 | 12.7 ±1.6 |  | 20 | 13.6 ±2.0 |
| 32 | 28 | 26 | 13.9± 2.1 |  | 30 | 12.6 ±2.3 |

T_D_, developmental temperature; Dpf, days post fertilization; SL, standard length

**Table S3.** Sampling information (per replicate) and morphometric measurements (mean standard length, SL) of zebrafish juveniles studied for heart morphometry.

| T_D_ (°C) | Dpf | R1 | |  | R2 | |
| --- | --- | --- | --- | --- | --- | --- |
|  |  | n | SL (mm) |  | n | SL (mm) |
| 24 | 35 | 10 | 12.9 ± 1.0 |  | 5 | 13.5 ± 1.6 |
| 28 | 28 | 5 | 11.9 ± 0.2 |  | 5 | 13.0 ± 1.2 |
| 32 | 28 | 4 | 11.6 ± 0.3 |  | 4 | 13.4 ± 0.2 |

T_D_, developmental temperature; Dpf, days post fertilization; SL, standard length

**Table S4.** Sampling information (per replicate) and morphometric measurements (mean standard length, SL) of zebrafish females and males studied for heart morphometry.

|  |  | Females | | | | |  | Males | | | | |
| --- | --- | --- | --- | --- | --- | --- | --- | --- | --- | --- | --- | --- |
|  |  | R1 | |  | R2 | |  | R1 | |  | R2 | |
| T_D_(°C) | Dpf | *n* | SL (mm) |  | *n* | SL (mm) |  | *n* | SL (mm) |  | *n* | SL (mm) |
| 24 | 280 | 5 | 33.0±0.7 |  | 4 | 32.0±2.2 |  | 5 | 32.0±0.7 |  | 4 | 31.9±1.4 |
| 28 | 280 | 4 | 32.6±0.7 |  | 4 | 31.4±2.6 |  | 5 | 31.7±1.2 |  | 4 | 32.1±1.8 |
| 32 | 280 | 3 | 31.9±1.7 |  | 4 | 33.0±1.9 |  | 5 | 31.8±0.8 |  | 3 | 31.1±1.4 |

T_D_, developmental temperature; Dpf, days post fertilization; SL, standard length

**Table S5.** Sampling information (per replicate) and morphometric measurements (mean standard length, SL) of zebrafish juveniles studied for swimming capacity.

| T_D_ (°C) | Dpf | R1 | |  | R2 | |
| --- | --- | --- | --- | --- | --- | --- |
|  |  | n | SL (mm) |  | n | SL (mm) |
| 24 | 77 | 9 | 16.9 ± 0.9 |  | 8 | 18.4 ± 1.1 |
| 28 | 70 | 7 | 16.7 ± 0.8 |  | 7 | 16.4 ± 2.0 |
| 32 | 64 | 7 | 15.7 ± 1.2 |  | 8 | 16.5 ± 0.9 |

T_D_, developmental temperature; Dpf, days post fertilization; SL, standard length

**Table S6.** Sampling information (per replicate) and morphometric measurements (mean standard length, SL) of zebrafish females and males studied for swimming capacity.

|  |  | Females | | | | |  | Males | | | | |
| --- | --- | --- | --- | --- | --- | --- | --- | --- | --- | --- | --- | --- |
|  |  | R1 | |  | R2 | |  | R1 | |  | R2 | |
| T_D_(°C) | Dpf | *n* | SL (mm) |  | *n* | SL (mm) |  | *n* | SL (mm) |  | *n* | SL (mm) |
| 24 | 148 | 5 | 23.9 ± 1.2 |  | 2 | 23.6 ± 0.8 |  | 4 | 22.7 ± 0.3 |  | 2 | 22.7 ± 0.4 |
| 28 | 141 | 5 | 24.1 ± 1.5 |  | 2 | 21.1 ± 0.3 |  | 3 | 23.7 ± 0.3 |  | 3 | 22.6 ± 0.9 |
| 32 | 136 | 3 | 26.7 ± 3.0 |  | 4 | 26.7 ± 1.6 |  | 3 | 23.5 ± 0.3 |  | 3 | 24.2 ± 1.1 |

T_D_, developmental temperature; Dpf, days post fertilization; SL, standard length

**Table S7.** Nucleotide sequences and gene accession numbers of primers used for Q-PCR.

| Gene  (Accession no.) | Forward primer  (5’ – 3’) | Reverse primer  (5’ – 3’) | Amplicon size (bp) |
| --- | --- | --- | --- |
| ***18S***  (BC155300.1) | CACTTGTCCCTCTAAGAAGTTGCA | GGTTGATTCCGATAACGAACGA | 91 |
| ***nppa***  (NM_198800.3) | ACAGCTCTGACAGCAACATGG | GTCTCTGTCCCAGGATGTGG | 154 |
| ***nfatc1*** (NM_001045159.1) | ATGTCCAACCCAAGTCTCATCA | GTGCCTATGAACAGCTGTAGGG | 147 |
| ***myh7***  (NM_001112733) | CAGAACCCTCCAAAGTTTGATAA | TATAGGCTACAACCACCTCCTGA | 192 |
| ***mybpc3***  (NM_001044349.2) | AACTGGAAGTTCGCCCTCAA | TCACACCATGGGGATGTGAG | 210 |

*18S*: 18S Ribosomal RNA; *nppa*: natriuretic peptide A (or *anf*); *nfatc1:* nuclear factor of activated T-cells 1; *myh7:* myosin heavy chain 7 (or ventricular myosin heavy chain, *vmhc*); *mybpc3*: myosin binding protein C, cardiac.
